# The Story of the Insane from Year to Year

**Published:** 1897-08-21

**Authors:** 


### THE STORY OF THE INSANE FROM YEAR TO YEAR.

Tenth Series.

### THE LEICESTER BOROUGH ASYLUM.

Here the numbers fell from 561 to 536. There were during the year 90 first admissions, 16 readmissions, 33 recoveries, and 37 deaths. The percentage of recoveries on admissions was 31·7, and that of the deaths on the average number resident was 7. Of the deaths 16 were caused by cerebral and spinal diseases, 3 by consumption, and 8 by general decay. Of the admissions, 12 owed their insanity to various moral causes, 11 to intemperance in drink, and 16 to hereditary influence. Dr. Finch had 112 epileptics under treatment, and not fewer than 10,538 fits were recorded. The Borough of Leicester patients have increased considerably of late years. In 1891 there were only 387 in the asylum, and now there are 527, showing an increase of 140 in six years, but Dr. Finch does not attribute this to an increase of insanity in Leicester. He thinks it is owing to an increase in the population, to the lowness of the asylum death-rate, and to the hopeless character of the cases admitted. At all events, the increase points to the necessity for more accommodation, and plans have been prepared for 360 pauper patients and 30 private patients. It is a pity the private patient's block is not to be for at least twice the number. The committee record their "appreciation of the highly satisfactory manner in which Dr. Finch and the officers of the institution have discharged their respective duties." The cost per head per week was 10s. 6⅝d.

### BARNWOOD HOUSE, GLOUCESTER.

There were 155 patients in residence on January 1st, 1896, and only 149 on December 31st. There were 22 first admissions, 7 readmissions, 13 recoveries, and 4 deaths. The recoveries stand at 44·8 per cent. on the admissions, and the deaths at 2·6 per cent. on the average number resident. Although the former is high and the latter low, no comparison can be made between a lunatic hospital and a county asylum because the conditions are totally different. When small-pox was raging in Gloucester, Dr. Soutar must have had a very anxious time, and it is very satisfactory to learn that the precautions he took to prevent the infection getting into Barnwood House were successful. The asylum or hospital is well known to be one of the best of its kind. It is liberally managed, and the amusements provided for the patients are numerous. The institution is to some extent a charity, and the committee say that "9 out of 13 applications for the maintenance of patients at reduced rates were granted, and it is estimated that the cost of maintaining 56 inmates who are in needy circumstances exceeded by almost £2,500 their aggregate payments." But this is rather a confused way of putting it, and what is wanted is a table showing the average cost per head of those patients on the charity and the payments respectively made by them. Dr. Soutar is to be congratulated. He has no charge attendant, on either side, of less than ten years' service.

### CUMBERLAND AND WESTMORLAND ASYLUM.

Here the numbers increased from 591 to 608. There were 129 first admissions, 33 readmissions, 76 recoveries, 21 discharged relieved, and 38 deaths. The recoveries stand at 46·9 per cent. of the admissions, and the deaths at 6·2 per cent. of the average number resident. Of the deaths 15 were caused by cerebral and spinal diseases, and 6 by consumption. Referring to the causes of insanity in the year's admissions, we find that 26 owed their attacks to such moral causes as domestic troubles, adverse circumstances, religion, and love; that in 23 the cause was intemperance in drink, and in 44 that it was hereditary influence. Recently the thyroid treatment has been advocated in certain forms of insanity; but Dr. Campbell, who has a sharp eye to the recovery rate in asylums, tells us it has failed in his hands, and that he cannot discover that it has "as yet left a marked impress on the general recovery rate of those asylums in which it has been used." Perhaps not, and we doubt whether any single line of treatment of any kind will ever leave such marks; but we believe we have seen much good follow the exhibition of thyroid extract in certain cases. Dr. Campbell deplores the tendency of the recovery rate in the Scotch asylums to become lower; but surely this chiefly depends on the nature of the admissions, and if we mistake not the readmissions are lower in Scotland than in England. We are pleased to notice that Mr. Todd, the clerk and steward, obtained his pension after thirty-three years' service. Dr. Campbell compares the duties of a medical superintendent with those of a bishop. We have never tried the latter, but we are certain that a medical superintendent "is incessantly harassed with a crowd of petty duties which leave no mark and win no credit." The visitors say that "the efforts made to effect the recovery of the patients by Dr. Campbell and the staff of officials at Garlands are well evidenced by the very satisfactory recovery rate and the low death-rate." A block for the treatment of private patients has been recently erected. This will prove a real boon to many patients of the lower middle class, and we are glad to learn that the minimum rate of charges has been placed low. The cost of maintenance is a fraction under 8s. 9d. per head per week.

